# Engagement over telehealth: comparing attendance between dialectical behaviour therapy delivered face-to-face and via telehealth for programs in Australia and New Zealand during the Covid-19 pandemic

**DOI:** 10.1186/s40479-023-00221-4

**Published:** 2023-05-20

**Authors:** Carla J. Walton, Sharleen Gonzalez, Emily B. Cooney, Lucy Leigh, Stuart Szwec

**Affiliations:** 1 Centre for Psychotherapy, Hunter New England Mental Health Service, 2300 PO Box 833, Newcastle, NSW Australia; 2 Department of Psychological Medicine, Wellington Medical School, University of Otago (Te Whare, Wānanga o Otāgo ki Te Whanga-Nui-a-Tara), Newtown, Wellington, New Zealand; 3 Yale University, New Haven, Connecticut, United States of America; 4 Data Sciences Unit, Hunter Medical Research Institute, Lot 1 Kookaburra Circuit, 2305 New Lambton Heights, NSW Australia

**Keywords:** Dialectical Behaviour Therapy, Telehealth, Telemedicine, Face-to-face, Covid-19, Attendance, Drop-out, Engagement

## Abstract

**Background:**

While the COVID-19 crisis has had numerous global negative impacts, it has also presented an imperative for mental health care systems to make digital mental health interventions a part of routine care. Accordingly, through necessity, many Dialectical Behaviour Therapy (DBT) programs transitioned to telehealth, despite little information on clinical outcomes compared with face-to-face treatment delivery. This study examined differences in client engagement (i.e. attendance) of DBT: delivered face-to-face prior to the first COVID-19 lockdown in Australia and New Zealand; delivered via telehealth during the lockdown; and delivered post-lockdown. Our primary outcomes were to compare: [1] client attendance rates of DBT individual therapy delivered face-to-face with delivery via telehealth, and [2] client attendance rates of DBT skills training delivered face-to-face compared with delivery via telehealth.

**Methods:**

DBT programs across Australia and New Zealand provided de-identified data for a total of 143 individuals who received DBT treatment provided via telehealth or face-to-face over a six-month period in 2020. Data included attendance rates of DBT individual therapy sessions; attendance rates of DBT skills training sessions as well as drop-out rates and First Nations status of clients.

**Results:**

A mixed effects logistic regression model revealed no significant differences between attendance rates for clients attending face-to-face sessions or telehealth sessions for either group therapy or individual therapy. This result was found for clients who identified as First Nations persons and those who didn’t identify as First Nations persons.

**Conclusions:**

Clients were as likely to attend their DBT sessions over telehealth as they were face-to-face during the first year of the Covid-19 pandemic. These findings provide preliminary evidence that providing DBT over telehealth may be a viable option to increase access for clients, particularly in areas where face-to-face treatment is not available. Further, based on the data collected in this study, we can be less concerned that offering telehealth treatment will compromise attendance rates compared to face-to-face treatment. Further research is needed comparing clinical outcomes between treatments delivered face-to-face compared delivery via telehealth.

**Supplementary Information:**

The online version contains supplementary material available at 10.1186/s40479-023-00221-4.

## Background

The global pandemic of Covid-19 thrust the mental health field into identifying an alternate way to deliver outpatient treatment that didn’t rely on face-to-face delivery. The use of videoconferencing technology to deliver care was already in place for some disorders [1] but not broadly utilized [2]. With the Covid-19 crisis, the options were either to pivot to telehealth or cease treatment to clients with severe mental health disorders. Therefore, many clinicians adapted to providing telehealth services during this time, even when empirical evidence was lacking. The rationale was that providing something was better than providing nothing [3] with a focus on aiming to maintain fidelity to the treatment model as much as possible [4]. The rapid transition to telehealth out of necessity highlighted how limited the data are on efficacy, engagement and retention of using telehealth for some psychotherapeutic treatments.

Telehealth has been defined as the provision of health care remotely by means of telecommunication technology [5]. It is also referred to as telemedicine and telepsychology. Telehealth has been recognized as a way to improve accessibility and cost-effectiveness [3]. This paper will focus on synchronous telehealth via videoconference, which refers to the delivery of services when both parties are conversing at the same time [6]. Prior to 2020, telehealth was primarily used in Australia for people residing in rural and remote areas rather than in metro and urban areas [7].

A decade of international research suggests that evidence-based psychological therapies can be delivered effectively and safely by videoconference for some of the common mental health conditions, such as anxiety, depression and PTSD for both adults and adolescents [2, 8–10] with systematic reviews showing outcomes comparable to face to face treatment delivery [3].

This is an important area of mental health research as telehealth increases access to effective psychological treatment. Major disparities in health internationally are largely a result of insufficient access to services [11]. Further, there are multiple benefits to delivery of treatment via telehealth – in addition to increasing access to treatment, for example, for those in rural and remote areas [12], it also provides flexibility to clients in reducing the time and cost of travel and eliminates the need for parking [13]. While this research was already happening prior to the pandemic, the impact of Covid-19 and the necessity for telehealth services has driven new studies on psychotherapy delivered via telehealth [14, 15].

Ease and equity of access is particularly important for treatments that require a high level of specialized training. Hence, it is surprising that few studies have examined the treatment of Borderline Personality Disorder (BPD) via telehealth, despite BPD being the personality disorder with the highest prevalence [16]. Fewer still report on the delivery of Dialectical Behaviour Therapy (DBT) via telehealth for clients with BPD and/or severe emotion dysregulation.

Standard DBT is an intensive, multi-modal treatment for complex, multi-diagnostic individuals with chronic suicidal and self-harming behaviour. It was originally developed for the treatment of BPD [17] and has now been expanded to the treatment of other disorders [18]. Standard DBT involves weekly group skills training, to acquire skills; weekly individual therapy to work on individual goals and help clients build ‘lives worth living’; phone coaching where therapists are available outside of regular session times to assist clients to generalise skills into their everyday lives and weekly consultation team meetings with both skills trainers and individual therapists to support each other to deliver adherent treatment and maintain motivation [17]. DBT is effective in reducing BPD severity, self-harm and improving psychosocial functioning [19] in multiple settings [20, 21]. However, to date there is no published research on the outcomes of DBT delivery via synchronous telehealth using teleconferencing.

Van Leeuwen et al. [22] published a comprehensive review of the availability, efficacy and clinical utility of DBT delivered via telehealth and reported “we did not find RCTs that tested the hypothesis that online or blended DBT is superior or at least equally effective as standard, face-to-face DBT (p.11)”. There are now several publications discussing how services running DBT programs adapted to using telehealth to deliver treatment [13, 23] in the context of the Covid-19 pandemic and there is evidence of an increase in online care in clinical practice [1, 24]. In addition, there is published research from survey results from clinicians and programs that had transitioned to delivery via telehealth. These papers report qualitative data on lessons learned in transitioning DBT to telehealth and how services and clinicians overcame (or didn’t overcome) barriers [25–27]. These published papers on ‘lessons learned’ call for empirical evaluation of the efficacy and effectiveness of DBT delivered via telehealth [25–27]. In summary, currently, the published research on DBT delivered via telehealth is largely limited to clinician feedback in qualitative studies.

There are a number of considerations that may limit the generalisability of telehealth research for the delivery of DBT. Most studies of telehealth have excluded people with suicidal behaviour [12], which is in part the population that DBT was designed for and frequently an inclusion criterion for the treatment [28–30]. Working alliance is particularly important in the treatment of people with severe emotion dysregulation and remote delivery may disrupt this by hindering the detection of subtle non-verbal communication [31]. Additionally, DBT has a group therapy component with limited data on its translation to telehealth delivery. In the few studies comparing face-to-face with telehealth group therapy, some have found comparable outcomes [32–34] and others have found better outcomes for in-person groups [35]. For DBT skills groups for depression, attendance was better for telehealth compared to a face-to-face group, however, those in the telehealth group reported less group cohesion than those in the face-to-face group [32]. There were no differences between the telehealth group and the face-to-face group in terms of clients’ relationship with the facilitator or the clients’ sense of their learning capacity.

As DBT was developed for the treatment of BPD and high-risk behaviour, clinicians have expressed concerns regarding client engagement via telehealth with the severity of their clients’ symptoms [8]. In addition, clinicians have expressed concerns that their clients will experience an increased sense of loneliness or abandonment; that in moderating an online group environment of clients who experience emotion dysregulation and are highly sensitive that there will be potential negative impacts of telehealth on group cohesion and they may be concerned about how effective their skills training is over telehealth. Recent qualitative research with adult clients who attended a DBT specific clinic via telehealth addresses some of these concerns. Dunn et al. [36] reported in a sample of 163 clients that their overall telehealth satisfaction rating was 82 out of 100, with the majority (80%) of those transitioning from in-person to telehealth reporting that their satisfaction with telehealth had stayed the same or improved across the transition. The majority (55%) indicated that telehealth became easier over time and that it increased their access to care (64%). However, in terms of sources of dissatisfaction with telehealth, being less connected to others was endorsed by 49% and feeling less connected to their therapist was endorsed by 36% of the sample. An Australian study evaluating telehealth satisfaction among 37 clients with BPD who received individual therapy sessions via telehealth with either mentalization-based therapy, DBT or a ‘common factors’ treatment approach found that 57% of clients appreciated not having the stress of travel, but 40% indicated that it was harder to make progress compared with face-to-face and 37% indicated that it was challenging to stay engaged and that it didn’t work as well as face-to-face [16].

Currently we are not aware of any published studies of quantitative data regarding Standard DBT delivered using synchronous telehealth, and insufficient research overall for telehealth to be considered evidence-based for suicidal ideation, self-harm or BPD [37]. We don’t know the clinical outcomes for people who participate in DBT via telehealth and whether they are similar to outcomes achieved when treatment is delivered face-to-face. At a basic level, in order to have the opportunity of having good clinical outcomes, clients need to have regular attendance. Apart from the lack of research on whether there are differences in clinical outcomes, there is no existing research on whether there are differences in attendance between delivery via face to face and delivery via telehealth.

This consideration may be particularly relevant for First Nations’ communities. Research investigating digital access for First Nations’ peoples is limited [38], however in both Australia and New Zealand First Nations’ people experience significant health and economic disparities [39]. In recently published research regarding barriers to engaging in DBT via telehealth [27], the most frequently reported barrier identified by DBT team leaders for First Nations’ peoples was lack of access to resources and privacy. Therefore, as part of addressing the gaps in healthcare, whilst we need to explore the use of technology, we need to ensure we are not exacerbating disparities [40].

In summary, the existing literature for other psychotherapies shows comparable outcomes when treatment is delivered via telehealth compared with face-to-face. This, in combination with the published research on transitioning DBT programs to telehealth, suggests that DBT could be effectively delivered via telehealth with good outcomes. However, some aspects of DBT limit our ability to generalize the findings from the existing telehealth literature and require research to determine whether clients are receiving the same quality of treatment on-line as when the treatment is delivered face-to-face.

This study was an opportunistic study conducted during the Covid-19 pandemic crisis that aimed to compare client engagement (i.e., attendance rates) when treatment was delivered over telehealth compared to face-to-face by DBT programs in Australia and New Zealand who transitioned to telehealth. In other words, we sought to empirically examine whether clients would attend treatment delivered via telehealth. We hypothesised that transition to delivery of DBT over telehealth would not be significantly different compared with face-to-face client attendance and drop-out rates.

Within our overarching aim, we had two primary and two secondary specific research questions:

Primary research questions:

1. Are attendance rates of DBT skills group therapy delivered via telehealth different as compared with delivery face-to-face?
2. Are attendance rates of DBT individual therapy delivered via telehealth different as compared with delivery face-to-face?

Secondary research questions:

3) Are client drop-out rates of DBT delivered via telehealth different as compared with delivery face-to-face?
4) Are First Nation peoples’ attendance rates and drop-out rates disproportionately impacted (i.e., higher) by telehealth service delivery compared to face-to-face service delivery?

## Method

### Ethics approval

The study was approved by Hunter New England Human Research Ethics Committee of Hunter New England Local Health District (Reference 2020/ETH02299) in Australia and University of Otago Human Research Ethics Committees in New Zealand (Reference HD20/109).

### Participants and recruitment

This study was cross-sectional. We invited leaders of comprehensive DBT programs across Australia and New Zealand who had pivoted to telehealth during the lockdown associated with the Covid-19 pandemic to provide de-identified clinic data of attendance and drop-out retrospectively. These were a subgroup of the respondents from an earlier study on the experience of providing DBT via telehealth, which provides further information about the methodology [27]. Team leaders of programs who had provided individual and skills training groups prior to the Covid-19 lockdowns and during the lockdown offered group skills training via videoconference telehealth and individual therapy (in either modality of face-to-face or telehealth) were asked at the end of the online survey if they were willing to be contacted regarding a further study. The participants were informed that the research team wished to compare client attendance levels of DBT delivered via telehealth vs. DBT delivered face-to-face. If they wished to participate in the further study, they were asked to write their name, role and contact details in a text box. There were 27 programs that were eligible for this study based on their responses in the earlier study. Of those 27 programs that were eligible for this study, 19 indicated a willingness to be contacted.

A member from the research team sent an email to those 19 people with an explanation about what the study involved and inviting them to participate. They were invited to provide the following to the research team to analyse: brief demographic information about their clients; and de-identified data from the time period of January 2020 to August 2020 regarding client attendance levels at group skills training and individual therapy and drop-out rates. In DBT, if a client misses either 4 sessions in a row of individual therapy or group skills training, they are unable to continue in the program and would be considered as ‘drop-outs’ [17].

Demographic information obtained included country where the program was run (Australia or New Zealand); geographical setting (urban (capital city), urban (other metropolitan area), rural (large rural area), rural (small or other rural setting), remote areas); service type: public health setting, private practice, non-profit and non-government organisations, private hospitals; program target age (adults, adolescents or both); indigenous status (yes or no).

In this article, we have chosen to refer to the participants of DBT as ‘clients’ for clarity for international readers. By ‘clients’, we are referring to the people that are fellow human beings that we are here to serve and to help them to live the lives that they would choose for themselves. We acknowledge that language is changing in this space and there are different preferences for those with lived experience, for example, to use the term ‘consumers’ or ‘service users’.

### Inclusion criteria

Eligible participants had to have: provided individual DBT and skills group therapy prior to Covid-19 lockdown; during the lockdown offered skills group therapy via video telehealth; and the research team had to be reasonably confident that the clients were receiving a standardised DBT program. Clients treated in these programs were required to have a diagnosis of BPD and/or significant emotion dysregulation. The research team confirmed with the DBT team leader that skills group therapy was offered weekly and that the clients were expected to attend weekly individual therapy at their service. Two clinics had incomplete or unreliable individual therapy data however the clients’ group data from these clinics were included because the DBT team leaders could confirm that these clients were receiving or had been scheduled for regular individual DBT at their clinics. A standard DBT program requires clients to attend individual and skills group training every week for a set number of weeks. Additionally, if a clinic only delivered skills group training and their clients received their individual therapy at a different clinic, the clinic was deemed not eligible for this study.

### Dates

Data was collected between March 2021 to October 2021 from historical attendance records for sessions attended between January 2020 and August 2020.

We used the following definition for the lockdown period with participants: “Your geographical area may or may not have had a formal lock-down: we are referring to the restrictions that applied to being able to provide face-to-face services in relation to preventing community transmission of COVID-19”.

### Data collection

Collection of de-identified attendance and drop-out data was sought across three time periods:

1. Time period 1: Primarily face-to-face service delivery, prior to moving to telehealth service delivery (approximately January to March 2020);

2. Time period 2: Treatment was delivered via telehealth when face-to-face groups could not be delivered and the majority of service provision occurred via telehealth due to concerns of community transmission of Covid-19 (approximately mid-March to May 2020);

3. Time period 3: Depending on the service, treatment was delivered either via face-to-face, or via telehealth or via a hybrid model where both face-to-face and telehealth were used (approximately June to August 2020).

The intended aim of the study design was to be an ‘ABA’ design (face-to-face > telehealth > face-to-face), however not all clinics returned to face-to-face service delivery during Time Period 3. Hence, some DBT clinics present an ‘ABA’ design, some clinics present an ‘ABB’ design (face-to-face > telehealth > telehealth) and some clinics present an ‘ABC’ design (face-to-face > telehealth > hybrid of face-to-face and telehealth).

De-identified data sought from DBT teams included the number of DBT therapy sessions attended per time period (if the client came for any amount of time for a group/individual session) and the number of scheduled DBT therapy sessions. This allowed us to calculate the attendance rate as the proportion of sessions attended out of the number of scheduled sessions. Information was also sought regarding the number of clients that discontinued therapy and the reason for this e.g. graduated from therapy, missed four consecutive therapy sessions. Clients who were considered to have dropped out of therapy, had to have missed four consecutive therapy sessions within the study time period. If the client dropped out of therapy after the study time period, they were not included as part of our ‘drop out’ data.

Dates provided by DBT team leaders allowed for all clinics to have eight-week time periods and their time periods were close together with minimal time gaps. Each clinic had their own unique time periods, as every clinic responded to the Covid-19 lockdown restrictions differently. However, the time periods for all clinics fell between January 2020 and August 2020. In a few instances, the third time period had to be adjusted by the research team because the clinic provided “Primarily telehealth” service delivery long beyond the first Covid-19 lockdown and they did not return to “Primarily face-to-face” until the end of 2020, which was out of the study’s scope. This also occurred for clinics who were in locations that had longer lockdowns and were unable to return back to “Primarily face-to-face” service delivery for an extended period. In these instances, a member from the research team adjusted that clinic’s third time period to start at the end of the second time period (8 weeks long), and that clinic’s third time period was re-classified to “Primarily telehealth”, to keep time periods for all clinics within the first seven months of 2020.

DBT team leaders received tailored spreadsheets for their clinics via email as well as a copy of a sample spreadsheet showing an example of how the data might look when entered (see Attachment 1). Instructions on how to complete the spreadsheets were included in the email.

One clinic struggled to complete their spreadsheet independently and sent the research team de-identified spreadsheets for the research team to collate on their behalf. If there were clients whose data appeared to be an extreme outlier, or there were missing data, the research team sought clarification and verification from the DBT team leader. In turn, that team leader contacted the clinician of that client directly. If the clinician identified as the contact person on the team did not respond to the team leader’s follow-up email, that information was considered missing.

We requested First Nations status data from clinics, to assess if first nations were disproportionately impacted by changes in service delivery during Covid restrictions. We also assessed for disproportionate impact of telehealth service delivery between clinics that were publicly compared to privately funded, and clinics that were in regional, rural or remote areas compared to clinics in urban centres or cities.

### Data analysis plan

To achieve sufficient power for this study, a sample size of 100 clients in each time period was required, assuming a 75% attendance rate in the first time period (i.e., primarily face-to-face service delivery), to detect an absolute 15% increase with 80% power and 5% significance. Assuming an average of 20 individuals per site, we needed data from a minimum of 5 participating sites.

Factors associated with attendances rates for group and individual therapy were examined using mixed effects logistic regression where clients were nested within sites to account for within-level similarities. Initially the variation at each level was assessed by fitting unconditional models (models with no predictors) and testing the significance of the variance using likelihood ratio tests. A random intercept at a participant level was included in subsequent modelling to adjust for correlation of outcomes within the participants.

Crude associations between attendance rates and therapy delivery mode were then assessed (crude model includes only delivery type) and a multivariable model was developed including all site-level variables.

The cohort in this study had a mix of early, middle and late-stage treatment dropout, however, the proportions of these are not known. As such, exploration of drop-out was restricted to just new starting clients.

All statistical analyses were programmed using SAS v9.4 (SAS Institute, Cary, North Carolina, USA). Significance was set as α = 0.05 a priori.

## Results

Of the 19 teams invited to participate, nine teams agreed and subsequently submitted data, three teams declined to participate, three teams were ineligible due to not providing both group and individual therapy to their clients, and four teams did not respond.

### Sample characteristics

De-identified data were collected from the nine DBT teams (n = 5 to 33 participants per team), which included a total of 143 clients for attendance at individual and group skills training and any persons that discontinued therapy between January and August 2020.

Descriptives of service provider characteristics are presented in Table 1.

**Table 1:** Service provider (site) descriptive statistics

Characteristic	Response	Total (n = 9)
Country	Australia	7 (78%)
	New Zealand	2 (22%)
Geographical setting	Urban (Capital City)	5 (56%)
	Urban (other Metropolitan Area)	3 (33%)
	Rural (small or other rural centre)	1 (11%)
Service Type	Public Health (i.e., Local Health District, District Health Board)	7 (78%)
	Private Practice	2 (22%)

The seven public health programs all indicated that they treat moderate to severe mental illness with their DBT programs having a focus on BPD and five of the programs required a diagnosis of BPD as part of the inclusion criteria of the service.

During data preparation, it was noted that there was complete separation between service type and First Nations status (no private practices recorded a clients’ First Nations status). Accordingly two separate multivariable models were developed, one including service type and one including clients’ First Nations status. One program was an adolescent program whose data were included in the overall sample with the data from the adult programs as otherwise the clients may have been identifiable. Hence, client population was discounted from the analysis as all but one service provided therapy to adults.

The only client level characteristic sought was First Nations status. 13 (9%) of the clients included in the overall data identified as First Nations Australian and New Zealanders, whilst 94 did not identify as First Nations people (66%) and there were missing data for 36 clients (25% of the sample). Missing data mean that the clinician from each program that extracted the data didn’t indicate either way for that consumer’s de-identified data. All clients who identified as First Nations people were seen in public health settings and spread across geographical settings.

### Number of clients who attended sessions across delivery modes

**Fig. 1:**
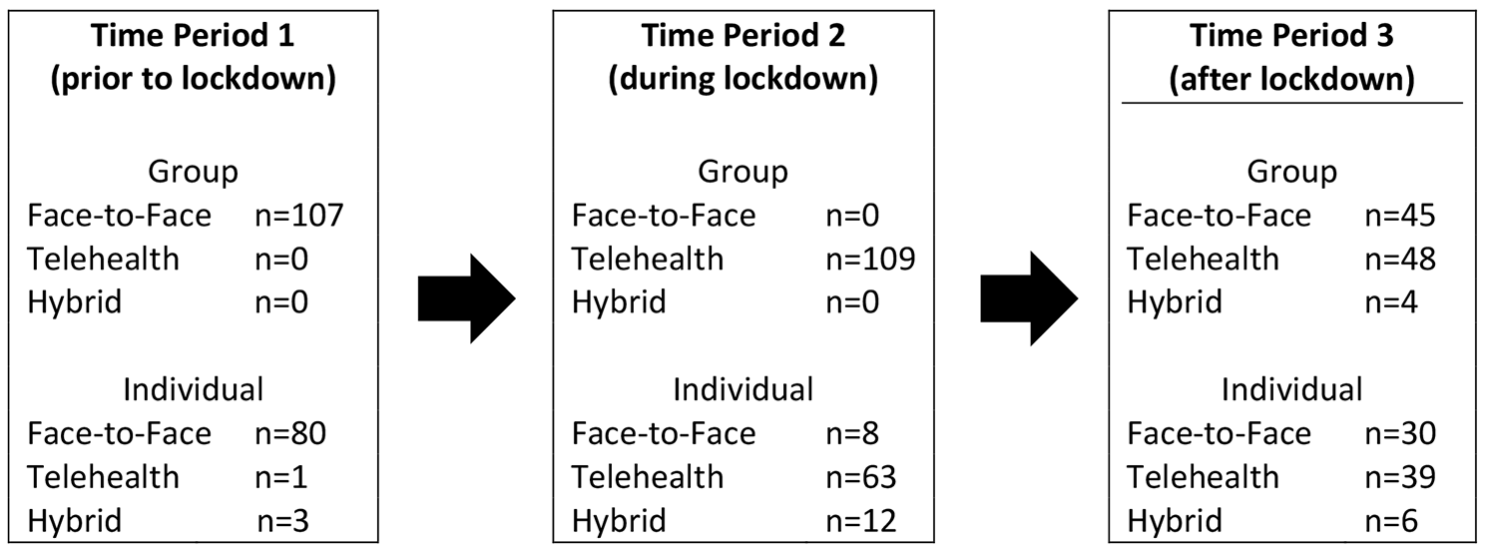
Attendance in each Time period by mode of delivery

There is a separation of delivery modes over the study, as presented in Fig. 1. 107 clients attended group skills training sessions in Time Period 1 (prior to the initial lockdown) delivered face-to-face and 109 clients attended group skills training sessions in Time Period 2 (during the Covid-19 lockdowns) delivered via telehealth. Group skills training sessions in Time Period 3 (once the initial lockdowns ceased) included both telehealth only (attended by 48 clients), face-to-face delivery only (attended by 45 clients) and a hybrid model involving both face-to-face delivery and telehealth delivery (attended by 4 clients). The small number of hybrid deliveries in Time Period 3 are too small to model separately (and combining them with another group would obfuscate the results) and as such are dropped from subsequent modelling.

The picture for individual sessions was slightly more mixed. There were missing data for two of the DBT programs for attendance at individual sessions (hence, 38 clients are not included in these numbers). Most individual sessions in Time Period 1 were delivered face-to-face (attended by 80 clients), however 1 client participated in therapy via telehealth and 3 clients attended a hybrid of telehealth and face-to-face. In Time Period 2, (during the Covid-19 lockdowns) most individual sessions were delivered via telehealth (attended by 63 clients) with 8 clients receiving treatment delivered face-to-face and 12 clients attended individual therapy with a hybrid of face-to-face and telehealth delivery. In Time Period 3 (once the initial lockdowns ceased), most clients attended therapy via telehealth (n = 39), 30 clients attended face-to-face and 6 attended via a hybrid model.

As the number of hybrid and telehealth sessions in Time Period 1, the number of face-to-face sessions in Time Period 2 and the number of hybrid sessions in Time Period 3 are too small to model separately (and combining them with another group would greatly reduce the interpretability of results), these were dropped from subsequent analyses.

### Association of group skills training delivery mode with attendance rate

The association of group skills training delivery mode with attendance rate was examined using mixed logistic regression modelling. Compared to face-to-face therapy (in Time Period 1), telehealth delivery (in Time Period 2) had lower odds of attending a group session (OR = 0.93) although the difference did not meet the threshold for statistical significance (p = 0.606).

No statistically significant differences were found in attendance rates for group therapy between delivery modes after adjusting for country, geographical setting, service type and First Nations status. As there were no clients of First Nations status seen in private practice, two separate multivariate models were run (one with country, geographical setting and service type, and one with country, geographical setting and First Nations status) to account for the different service type.

### Association of individual therapy delivery mode with attendance rate

The association of individual therapy mode with attendance rate was also examined using mixed logistic regression modelling. Compared to face-to-face therapy (in Time Period 1), telehealth delivery (in Time Period 2), had higher odds of attending an individual therapy session (OR = 1.53), however, the difference did not meet the threshold for statistical significance (p = 0.057).

No statistically significant differences in individual therapy attendance rates were found between delivery modes when adjusting for service level characteristics or First Nations status.

Table 2 shows descriptive statistics (means and medians) of the proportion of group and individual sessions attended in each time period.

**Table 2:** Comparison of the proportion of sessions attended by delivery mode

Characteristic	Response	Face-to-face Time period 1	Telehealth Time period 2	Face-to-face Time period 3	Telehealth Time period 3
Group attendance rate	Mean (SD)	0.74 (0.24)	0.76 (0.30)	0.72 (0.28)	0.85 (0.17)
	Median (Q1, Q3)	0.78 (0.57, 1)	0.88 (0.63, 1)	0.75 (0.5, 1)	0.88 (0.75, 1)
Individual attendance rate	Mean (SD)	0.82 (0.19)	0.86 (0.25)	0.82 (0.25)	0.86 (0.22)
	Median (Q1, Q3)	0.88 (0.73, 1)	1 (0.83, 1)	0.88 (0.8, 1)	1 (0.75, 1)

### Descriptive analyses of those who discontinued therapy

Clients discontinued therapy for a range of reasons, including successfully completing treatment (referred to graduating for treatment); graduating from treatment; not being able to continue therapy over telehealth; and dropping out of therapy. 24 clients graduated during the study period. Of those, 13 graduations occurred in Time Period 1, 11 occurred in Time Period 2 and 1 occurred in Time Period 3. No statistical analyses were possible due to the limited numbers of clients graduating.

One of the DBT programs was not included in the drop-out analysis as numerous exemptions to the ‘4 miss rule’ were made during Covid-19 lockdown. 29 clients started group skills training and 21 clients commenced individual therapy during the time of the study. Due to limited numbers of clients starting therapy during the course of the study, no statistical analyses were possible and only numbers of patients dropping out by time period and delivery mode are reported. Whilst attendance rates for group and individual sessions may be different, dropping out of treatment in DBT means dropping out of the program altogether, hence, the numbers reported are for dropping out of therapy across individual and group therapy.

Three of the new starting clients dropped out of therapy. These were all clients who had started group skills training during the study timeframe and they dropped out of telehealth delivered therapy in Time Point 3.

## Discussion

In a naturalistic study, we sought to identify whether rates of face-to-face attendance at DBT group skills training and individual therapy were comparable to rates of attendance via telehealth during the Covid-19 pandemic. In addition, we sought to compare whether there were differences in drop-out rates from DBT group skills training and individual therapy between treatment delivered face-to-face and treatment delivered via telehealth. Lastly, we were interested in whether First Nation clients’ attendance rates and drop-out rates were disproportionately impacted by telehealth service delivery compared to face-to-face service delivery.

There were no significant differences between attendance for either DBT skills training groups or individual therapy sessions between face-to-face and delivery via telehealth during the Covid-19 pandemic. The drop-out data numbers were too small to accommodate statistical analyses. There were no significant differences between attendance rates for First Nation clients between face-to-face and telehealth delivery.

The study focused on testing a common clinician assumption and concern that clients with severe mental health symptoms will not engage as much on telehealth as they will face-to-face and hence, that it may be inappropriate to offer DBT over telehealth because of concern regarding poor attendance. This study’s findings provide important preliminary data on the potential for telehealth therapy options to increase access and equity to mental health treatment for this vulnerable group whose treatment options are already limited.

Given the number of programs that are continuing to deliver DBT via telehealth (out of choice or necessity), it is pleasing to know that clients across Australia and New Zealand in this study received a comparable amount of treatment regardless of attending face-to-face or via telehealth during the Covid-19 pandemic. This indicates that on the whole, clients were willing to engage with treatment via telehealth during this time.

While the literature is still emerging in this area, our findings reflect positive attendance rates previously found for clients engaging in telehealth therapy [14, 32]. While our study found no difference in attendance rates between face-to-face and telehealth therapy sessions, Silver and colleagues reported a statistically significant improvement in attendance at a primary health care facility when the service transitioned to exclusively telehealth sessions because of Covid-19 risk. While interesting, the findings are limited as the sample was of 8 clinicians who worked at the one hospital who tracked their clients’ rates of missed therapy appointments. Some of Silver and colleagues’ hypotheses for this were improved convenience, decrease in certain barriers such as transport issues, telehealth provided some distance between client and therapist which may be comforting for clients who struggle with intimacy, and therapists taking initiative to start the session as they were the ones to make the phone call and send the video link and clients may have enjoyed this “active pursuit”. Lopez and colleagues [32] found significantly better attendance for a telehealth DBT group compared to a face-to-face group. They also hypothesized that telehealth may have reduced access barriers.

Our study only captured attendance data, and not the lived experiences of the clients. However, available data from qualitative studies of both clients with severe emotion dysregulation [36] and other clinical populations [32, 41–43] complement the finding of comparable attendance rates between telehealth and face-to-face DBT. Taken together, these qualitative studies indicate that even though clients would prefer a face-to-face group, if possible, telehealth is preferable to nothing. As such, it is possible that the convenience of a telehealth group outweighs both the negatives of telehealth and the positive aspects of in-person groups that are missed over telehealth, explaining a net equivalence in attendance rates between these two methods of delivery.

Within our sample, people who identified as First Nations’ peoples of Australia and New Zealand did not appear to be disproportionately impacted by telehealth service delivery. These groups were just as likely to attend telehealth sessions as face-to-face sessions compared with individuals who didn’t identify as First Nations people. Even though the research is limited, this finding appears to be consistent with some of the findings about digital access in this group reported by Rennie et al. [38] who compared data from the Australian Digital Inclusion Index (ADII), the National Aboriginal Torres Strait Islander Social Survey (NATSISS) and the Census of Population and Housing. They reported that the digital access gap between those who identify as Aboriginal and Torres Strait Islander in Australia and those who don’t identify who live in urban areas is shrinking and that individuals who identify as First Nations’ peoples reported having more positive attitudes towards technology than non-identifying. However, they are also more likely to be mobile-only users and may have disadvantages in regards to this. Our study did not assess whether clients were attending their appointments via a laptop, computer or mobile phone. Therefore, while our results are promising for people living in urban settings, future research should consider the methods in which clients’ attend appointments and whether this affects outcomes.

Certain contextual factors may have enhanced telehealth attendance, independent of the desirability of this delivery method. The telehealth time period occurred when geographical areas were in lock-down. Many of the clients would have been at home without other occupational or social demands hence it may have been easier to attend in this time period. Attending group and individual therapy may have given them something to do as many people reported being bored during this time [44] as well as helped them maintain their connection with others in a time of crisis [14]. In addition, clients’ options were typically binary (telehealth or nothing) and may not reflect telehealth uptake during a context when it is optional.

Conversely some factors may have had a negative impact on attendance via telehealth. Teams had not previously delivered DBT skills training via telehealth. Many clinicians were apprehensive about delivering DBT via telehealth and were resistant to change, which may have impacted on their enthusiasm and the quality of the treatment being provided [27, 45]. Some research has shown that clinicians have expressed a preference for face-to-face treatment delivery [3] and a reluctance to use telehealth for a number of reasons [8]. These reasons include: lack of training, and concerns about efficacy, safety, privacy, and ability to navigate technology, which may all impact on attendance. Many clinicians and teams were learning lessons about how to deliver therapy via telehealth as they were delivering it and delivery was often beleaguered by technological challenges, and stressful [13, 25–27] and therefore unlikely to be a polished product. There are likely to be a range of factors that influence clinician attitude towards telehealth including familiarity and experience with it, treatment model, diagnostic group, organisation support and available resources. For example, in our experience, doing groups via telehealth is much easier with some teleconference platforms that have greater functionality than it is with other teleconference platforms with lesser functionality.

The results are aggregated across all clients, and therefore don’t illuminate at an individual level if some clients are more likely to attend on face-to-face and some more likely to attend on telehealth. Dunn et al. [36] asked clients in their DBT clinic once Covid-19 was no longer a factor what mode of delivery they would prefer. For individual therapy, 34% chose face-to-face, 30% chose a combination of both, 24% chose telehealth and 12% were unsure. For group skills training, 33% chose telehealth, 30% chose face-to-face, 26% chose a combination of both and 10% were unsure (1% did not respond). Based on the responses from that study, it seems likely that some clients may have been more likely to attend therapy on telehealth than face-to-face and vice versa for other clients. Averaging attendance data obscures this important consideration. In addition to this possible selection bias issue, clients who started DBT during time period 2 may have had a preference for telehealth. These clients started DBT during Covid-19 lockdowns and were aware that telehealth therapy was the only option available. Clients that prefer face-to-face may have chosen to wait to start DBT until it was offered face-to-face again in time period 3 or beyond the study period.

Unfortunately, we did not have sufficient information to look at significant differences in drop-out rates comparing face-to-face delivery and telehealth or transitioning between the modalities. This would require a much larger sample to understand whether there are significant differences between DBT delivered via telehealth and via face to face for clients graduating from therapy or dropping out of therapy and whether clients may be more likely to drop out earlier or later in treatment when delivered via telehealth. The DBT program that the first and second author work in has been moving between periods of treatment delivery face-to-face and via telehealth for nearly three years since early 2020 in line with increased risk of transmission associated with the Covid-19 pandemic. Anecdotally, we have noticed greater rates of drop-out when clients commenced on telehealth and the program then transitioned back to face-to-face. This requires further examination with larger samples.

Another limitation of this study is that the data were collected over a brief time period (approximately 2 months for each time period). As such, we don’t know how generalizable these results are to treatment after the first peak of the pandemic. This study relied on the accuracy of recording of attendance and clinicians may have had different approaches to how they calculated missed and scheduled appointments per week (see Appendix 1 for the standardised spreadsheet used by team leaders). The research team dedicated time to corresponding with team leaders to get as much clarification as possible on this to ensure the data were as clean and representative as possible.

Whilst five of the programs required a diagnosis of BPD, we do not know with certainty about diagnoses for the other programs, only that the clients were considered suitable for DBT. We also do not know about the fidelity to the model of the treatment programs that were involved, nor what the standard clinical outcomes were of the programs involved in this study. Hence, we were comparing whether overall there was a reduction in attendance when clients who were considered suitable for DBT were participating via telehealth in contrast to when they had attended face-to-face. Future naturalistic research would be strengthened by investigating attendance and drop-out over longer period with programs that are certified allowing the assumption of a minimum level of fidelity.

As only one adolescent team was included, we could not examine whether there are differences between adolescent programs compared with adult programs. A recent review by Reis et al. [46] did not find any studies of online intervention for adolescents with personality disorders, hence, more research is needed in this area. Given the well-documented facility with online solutions experienced by digital natives, it may well be that patterns of telehealth attendance for adolescents may be very different from their adult counterparts.

This study compares attendance levels in telehealth and face-to-face DBT for those clients who were able to engage remotely. Barnett et al. [3] in their umbrella discussion of systematic reviews note that researchers did not generally report what percentage of clients were excluded because they did not have the resources to engage in telehealth. This was not assessed in our study, but in Cooney et al.’s [27] qualitative study practical technological issues and resource deficits for clients and clinicians were one of the most-cited barriers to providing DBT over telehealth. Without attending to the lack of resources to engage in telehealth, excluding people may result in the exacerbation of existing inequalities.

This study has a number of strengths. It was sufficiently powered to identify differences based on attendance rates. Despite the possible likelihood that some clients may have a preference for telehealth or face to face, we believe the risks of selection bias were low given that we collected service level attendance data for all clients. In addition, the programs contributing data are relatively homogenous in terms of the structure and represent a range of programs across Australia and New Zealand rather than from a single service.

To our knowledge, this is one of the first studies to provide quantitative data for people with severe emotion dysregulation who were enrolled in a DBT program and participated via telehealth. Ultimately, we need data from randomised clinical trials comparing clinical outcomes between those who attend face-to-face and those who attend on telehealth, however, this is beyond the scope of what we could measure given that this was an opportunistic study conducted during the Covid-19 pandemic. This is an important area of future research. It would also be helpful to examine telehealth vs. face-to-face attendance for adolescents specifically.

In the absence of empirical research regarding the use of DBT delivered via telehealth, when the Covid-19 pandemic began and lockdowns prevented delivery of face-to-face treatment, DBT programs that transitioned to telehealth needed to make a number of assumptions. One of them was that delivery of the treatment via telehealth would be better than no treatment at all. Whilst there are no trials comparing those two options, anecdotally from the qualitative research that has been conducted [13, 25–27], this study contributes to the evidence for utility in delivering DBT via telehealth.

Comer [2] suggests that with the widespread uptake and acceptability among both clinicians and clients that the use of telehealth will outlast the Covid-19 pandemic and become the dominant mode of mental health delivery. This is consistent with the data from a survey in the US of mental health clinicians and organisation who reported a high likelihood that telehealth would continue post-pandemic [47]. However, as we move out of the Covid-19 pandemic, ethically, we need to try and determine whether delivery of treatment via telehealth for people with a diagnosis of BPD or severe emotion dysregulation is equivalent to outcomes achieved where the therapy is delivered face-to-face or in what situations it is a preferred or less preferred option in order to be able to be transparent with clients regarding the state of the evidence. It is not ethical to fall into a method of delivery that yields poorer outcomes, through convenience. In their comprehensive review of the availability, efficacy and clinical utility of DBT delivered via telehealth Van Leeuwen et al. [37], suggest a return to face-to-face contact as soon as is possible, with a shift to DBT delivered via telehealth only being justified if it is the only way to get an evidence-based treatment like DBT to patients that need it. To build on the findings of this paper and our current knowledge about DBT delivered via telehealth, it would be valuable to track attendance and drop-out rates of clients enrolled in face-to-face and telehealth DBT programs over a longer timeframe with a larger sample.

## Conclusion

In this study, DBT programs across Australia and New Zealand provided de-identified data regarding attendance rates and drop-out rates for treatment provided via telehealth and face-to-face over a six-month period. We did not find any significant differences between attendance rates between face-to-face and telehealth DBT therapy sessions for individual therapy or group therapy. Clients, regardless of First Nations status, were just as likely to attend telehealth DBT therapy sessions as they were face-to-face sessions. Unfortunately, it was not possible to compare dropout rates due to the small number.

Anecdotally, DBT has continued to be delivered over telehealth in many services, particularly those in the private sector. Further research is needed into the clinical outcomes of those that receive DBT delivered via telehealth compared with face-to-face treatment delivery once lockdowns and restrictions on movement cease to ensure the outcomes are comparable, especially for clients struggling with severe emotion dysregulation and suicidal and self-harming behaviours.

## Electronic supplementary material

Below is the link to the electronic supplementary material.

Supplementary Material 1

## Data Availability

The dataset supporting the conclusion of this article are held by the authors and will be made available upon justified request.

## Abbreviations

DBT: Dialectical Behaviour Therapy
BPD: Borderline Personality Disorder.
